# Barriers and Enablers to Capsule Sponge Screening Among People With Heartburn: A Community‐Based Qualitative Study in the UK

**DOI:** 10.1111/hex.70919

**Published:** 2026-10-15

**Authors:** Ninian Schmeising‐Barnes, Emma Lidington, Elisavet Moschopoulou, Daksha Trivedi, Jo Waller

**Affiliations:** ^1^ Centre for Cancer Screening, Prevention and Early Diagnosis, Wolfson Institute of Population Health Queen Mary University of London London UK; ^2^ School of Health, Medicine and Life Sciences, Centre for Research in Public Health and Community Care University of Hertfordshire Hatfield England

**Keywords:** acid reflux, attitudes, Barrett's oesophagus, indigestion, intention, non‐endoscopic, oesophageal cancer

## Abstract

**Introduction:**

Non‐endoscopic capsule sponge devices are a promising new technology. They could potentially improve oesophageal cancer outcomes by detecting and monitoring Barrett's oesophagus and identifying dysplasia early to allow timely treatment. Trials assessing their clinical utility are underway.

**Objective:**

This study sought to understand potential barriers and enablers to capsule sponge screening among people with heartburn.

**Methods:**

Forty semi‐structured interviews were conducted between October 2024 and April 2025 with men aged 55–79 and women aged 65–79 taking regular reflux medication. Participants were purposively sampled. Transcripts were analysed using Thematic Framework Analysis.

**Results:**

Participants were generally enthusiastic about the test, but not all felt willing or able to take part. Key factors influencing their attitudes are described in three overarching themes. First, participants described how they navigate reflux‐related care and negotiate this with healthcare professionals. They considered how capsule sponge screening would fit into this collaborative model. Second, participants showed an aversion to uncertainty, noting that capsule sponge screening could both create and reduce uncertainty about their health. Third, participants' understanding of their candidacy varied based on risk perceptions, self‐efficacy and views about acting on test results. Some expressed a sense of being less ‘worthy’ candidates than others.

**Conclusion:**

Overall, the capsule sponge test screening appealed to many due to its quick, simple and minimally invasive approach. Nevertheless, concerns about responsibility for reflux care, candidacy and uncertainty will likely shape attitudes and intention towards the test and should be key considerations if this test is implemented.

**Lived Experience or Public Contribution:**

Three individuals with lived experience of reflux, Barrett's oesophagus and oesophageal cancer were involved in the design of this study and associated materials.

## 1 Introduction

Oesophageal carcinoma is often detected late due to vague, late‐presenting symptoms and survival is poor [1]. One subtype, oesophageal adenocarcinoma (OAC), has recently increased in prevalence in high‐income countries, including the United Kingdom [2]. Barrett's oesophagus (Barrett's) is a pre‐cancerous risk factor for OAC, where the cells lining the oesophagus change shape [3]. Both OAC and Barrett's are more common among people with long‐term acid reflux [4]. Those with Barrett's are offered regular endoscopic monitoring so that high‐grade dysplasia and cancer cells can be detected early and treated if necessary [5]. Currently, Barrett's is diagnosed via upper endoscopy. Endoscopy is expensive, labour‐intensive, invasive and under high demand [6, 7], meaning it is not currently recommended for those with reflux, unless they show three or more other risk factors [5]. Estimates suggest that the population prevalence of Barrett's in high‐income countries is ~2% [8], yet only one in five cases are thought to be diagnosed [9, 10], leading to inadequate monitoring.

To increase capacity for Barrett's screening, novel, non‐endoscopic methods are being trialled in the United Kingdom [11], including swallowable cell collection devices such as the Cytosponge™ [6]. These tests, also known as capsule sponge tests, can be used to screen those with reflux for Barrett's. During the test, the patient swallows a small capsule attached to a thread. Once in the stomach, the outer coating dissolves, releasing a small sponge. A trained healthcare professional then pulls the thread to retrieve the sponge, collecting a sample that is analysed for signs of Barrett's. If the test indicates Barrett's, patients are referred for diagnostic endoscopy [6].

If shown to improve oesophageal cancer outcomes, the success of any targeted capsule sponge screening programme would depend on good uptake in the eligible population. Recently, the NHS has announced plans to use capsule sponge tests within a pilot heartburn health check service that could be rolled out more widely if proven successful [12]. In addition, the UK National Institute for Health and Care Excellence (NICE) is currently performing an extensive appraisal of this technology to inform potential implementation [13].

Screening uptake is influenced by a range of barriers and enablers [14]. Per the Integrated Screening Action Model (I‐SAM), barriers and enablers operate at an individual or environmental level [15]. Individual influences include motivation, self‐efficacy, and practical barriers. Environmental influences include access to healthcare, social norms and how the test is offered. Barriers and enablers to screening differ between individuals, for example, based on physical disability [16] or ethnicity [17], and between screening programmes [15, 18, 19, 20].

Most research investigating public attitudes towards capsule sponge screening has been embedded within clinical trials [21], where the test has been rated as highly acceptable [22, 23]. Despite this, trial participation is typically low (~25%) [22], which could reflect barriers to uptake. Studies exploring capsule sponge screening acceptability have suggested that limited awareness of Barrett's [24, 25], difficulty judging personal risk [22], discomfort [22, 25] and procedural concerns [26] may impede participation. Enablers include low test invasiveness, practicality and cost‐effectiveness [21, 26, 27, 28, 29]. Despite some research being conducted, the samples of such studies have been limited in that they either included those who had already had the test, indicating their perceived acceptability of it, or participants recruited from online market research panels, who generally held positive intentions towards the test. As such, the barriers and enablers to capsule sponge screening in more diverse samples, especially those less positively inclined towards the test, have not yet been explored.

This study sought to gain an in‐depth understanding of potential barriers and enablers to capsule sponge screening, with a particular focus on individuals less inclined to have the test. Furthermore, it aimed to understand factors influencing intentions and key considerations for implementation.

## 2 Methods and Materials

This study was approved by the Queen Mary University of London Research Ethics Committee (QME24.0465) and is reported according to the COREQ checklist (see Supporting Information S1: Supporting Materials 1). The full dataset is available upon request.

### 2.1 Design

Semi‐structured interviews were used to encourage in‐depth discussion and afford flexibility to cover emerging topics.

### 2.2 Participants

Inclusion criteria reflected current UK trials for capsule sponge screening: men and non‐binary individuals aged 55–79 and women aged 65–79, living in the United Kingdom, and taking reflux medication at least twice weekly for the past 6 months.

We purposively recruited participants based on capsule sponge screening intention, gender and education. We aimed for a 50:50 split based on gender, education above and below degree level, and between intenders and non‐intenders. Non‐intenders were those who said they would ‘probably not’ or ‘definitely not’ accept an offer of capsule sponge screening (when provided with brief information), or if they said, ‘don't know’. We recruited across all four UK nations, ensuring > 60% lived outside London, and that some were from rural areas.

Those diagnosed with oesophageal cancer or Barrett's, or who had experience of capsule sponge screening, were not eligible for this study.

### 2.3 Recruitment

Participants were recruited through community groups and UK employers from October 2024 to April 2025. Several advertising methods were used, including posters, social media and in‐person attendance at the organisations. We also invited participants through Heartburn Health, a UK‐based cohort of people with reflux [30], to bolster recruitment of male non‐intenders who were difficult to identify via other routes.

### 2.4 Procedure

Interested participants completed a pre‐screener questionnaire to determine their eligibility (see Supporting Information S1: Supporting Materials 2).

All interviews were conducted by NSB via Microsoft Teams, telephone or in‐person. In‐person interviews were conducted at the university, participants' homes and community locations. A semi‐structured topic guide was developed based on previous literature, the I‐SAM and discussions with patient and public involvement (PPI) and academic collaborators (Supporting Information S1: Supporting Materials 3). Participants received information about capsule sponge screening, including a short video (Supporting Information S1: Supporting Materials 4). Following the interview, participants were debriefed, including being reassured about their low risk of oesophageal cancer and Barrett's. Interviews were recorded and transcribed verbatim. Participants received a £25 shopping voucher for taking part.

### 2.5 Patient and Public Involvement

Three PPI contributors provided feedback on the study procedures, topic guide and documents. One contributor participated in a pilot interview. PPI feedback led to changes in the wording of materials and the information provided in the interviews. A full PPI statement, in line with the GRIPP2 Short Form checklist, is available in Supporting materials.

### 2.6 Framework Analysis

Data was analysed using Thematic Framework Analysis [31] in NVivo 14. The coding framework is available in Supporting Information S1: Supporting Materials 5. This approach follows six steps (Table 1):

**Table 1:** Thematic framework analysis process.

Step	This study
Familiarisation with the transcripts	NSB checked and anonymised the transcripts, wrote notes and summaries. The transcripts were not shared with participants for comments or correction.
Initial coding	NSB conducted line‐by‐line open coding using an inductive approach on a subset of transcripts.
Framework creation	NSB categorised codes, incorporating information from field notes. NSB wrote definitions and descriptions for each category. Example quotes were presented to the other co‐authors.
Applying the framework	NSB conducted line‐by‐line coding on the remaining transcripts. The framework was refined iteratively in meetings with co‐authors.
Charting	NSB created a matrix and wrote summaries.
Interpretation	NSB identified patterns across the matrix to identify latent themes.

### 2.7 Reflexivity

All co‐authors are female and have expertise in cancer screening, conducting interviews and qualitative data analysis. NSB has an MSc in Health Psychology. All other authors have a PhD in Health Psychology or Health Sciences. NSB is a PhD student, EM is a postdoctoral research associate, EL is a senior research fellow, and DT and JW are professors of applied health research and behavioural science respectively.

We adopt a critical realist perspective, recognising that people construct their own understandings of healthcare, but are influenced by structural, cultural, economic and political factors. We believe that knowledge about public health interventions is accessed through participants' reported cognitions, emotions and behaviours, mediated through social determinants of health and contextual factors. We take an empiricist perspective to data collection and analysis but recognise that both participants' narratives and researchers' interpretations are influenced by social constructs and external factors, influencing the knowledge created. Throughout data collection and analysis, NSB kept a reflexive journal to reflect on how her own experiences and assumptions may have influenced her approach and interpretation. This practice was used to challenge assumptions and prompt the exploration of alternative perspectives. A reflexivity statement is available in Supporting Information S1: Supporting Materials 6.

## 3 Results

### 3.1 Sample Characteristics

We conducted 42 interviews with 20 men and 22 women aged 55–79. Two male participants were excluded post‐interview due to ineligibility and suspected fraudulent participation (further detail is available in Supporting Information S1: Supporting Materials 9), meaning 18 men were included in the analysis.

Participants represented a good spread of gender, age, education, geography and intention (Table 2). Compared to intenders, a larger proportion of non‐intenders were female and had below‐degree‐level qualifications. All non‐intenders were in the older age group (65–79 years old).

**Table 2:** Participant sociodemographic characteristics and intention to accept an offer of capsule sponge screening prior to the interview (*n* = 40).

GENDER (AGE)	
Men (55–64)	7
Men (65–79)	11
Women (65–79)	22
Capsule sponge screening intention	
Yes, definitely	14
Yes, probably	11
No, probably not	9
No, definitely not	2
Don't know	4
Location^a^	
London	9
West Midlands	1
East of England	9
Southwest England	1
Yorkshire	1
Northern Ireland	5
Wales	7
Scotland	7
Rurality^b^	
Urban	25
Rural	15
Education^a^	
No formal qualifications	4
GCSEs or equivalent	6
AS, A‐level or equivalent	3
NVQ up to level 3 or equivalent	2
Apprenticeship	2
Qualification at degree level or above	17
Any other qualifications or equivalent unknown	4
Prefer not to say or blank	2
Disability^a^	
Yes	7
No	33

^a^ Measured using 2021 census items.

^b^ Measured using the Office for National Statistics 2011 Urban/Rural Indicator (postcode data).

Interviews ranged from 34 to 77 (mean: 50) minutes.

### 3.2 Themes

Three latent themes were identified [1]: A collaborative affair: navigating reflux‐related care [2]; Coping with uncertainty and [3] Proving yourself as a worthy candidate for capsule sponge screening. A detailed description of each theme is provided below, and additional illustrative quotes are available in Supporting Information S1: Supporting Materials 7. Information provided in square brackets indicates the participants' characteristics, for example: [W, non‐intender, degree+] means the participant is a woman, non‐intender with educational qualification above degree level.

#### 3.2.1 A Collaborative Affair: Navigating Reflux‐Related Care

Participants described differing beliefs about who is responsible for managing their reflux and related care. Some wished to have a more active role in their reflux care that spanned across the capsule sponge screening pathway—from symptom management and risk awareness to views on the screening procedure and acting on the results. Others expected healthcare professionals to direct reflux‐related care.

#### 3.2.2 Symptom Management

Many felt that their symptoms stemmed from their own behaviours, such as ‘overindulging’ or eating foods that can trigger reflux and attempted to control this through lifestyle change.

I've tried to reduce the amount that I eat, tried to eat at different times of the day, I tend to try and sleep a bit more upright, and I tend to sleep on my left side because I know that that sort of helps… but then I have possibly overindulged … I think *oh, you're going to pay for this later*.

(P17, W, non‐intender, degree+)

Some did not see reflux as a serious health condition, especially if it was well managed with medication or lifestyle change and didn't see the need to consult a healthcare provider about it. Others described deliberately avoiding healthcare professionals to prevent becoming ‘tied to taking’ medication.

If I can get off the medication, that's a good sign to me if I can control it with diet or something else….

(P20, M, intender, <degree)

Despite personal efforts, many eventually turned to healthcare professionals for advice about managing their reflux. Often this resulted in participants being prescribed proton pump inhibiting (PPI) medication. Some described ‘blindly’ taking PPI medicines for decades, per their GP's advice, and were angry and disappointed when they learnt about the potentially harmful effects of long‐term use.

You take a long‐term medication not realising that it's setting you up for all sorts of other things later in life… you're not supposed to take it for 27 years… Nobody ever addressed that with me.

(P11, M, intender, degree+)

Participants described how capsule sponge screening could be used to confirm that their reflux management techniques were working and that their reflux was not causing them significant health problems.

What it might do is give me some comfort that I can live with the reflux, and I know or don't know what damage it's doing, whereas at the moment, I'm ignorant to what damage it's doing.

(P2, M, intender, degree+)

#### 3.2.3 Reflux Risk Awareness

Most participants had never heard of Barrett's and were unaware of the increased cancer risk associated with long‐term reflux. Some felt that healthcare professionals should make their patients aware of the risks associated with reflux but also recognised that this is only possible if individuals consult their GP about their reflux, which is often not the case.

When discussing participants' perceptions around the risk associated with their reflux, some were confident to estimate their risk based on their symptom experience and personal research. Others lacked confidence to estimate their own risk and said they would seek their healthcare professional's judgement for recommendations about screening.

Well, the healthcare professionals… ask them advice about whether there's an association or anything … sort of recommended if you like.

(P6, W, intender, >Degree)

#### 3.2.4 Capsule Sponge Screening Eligibility and Procedure

Some were concerned that they would not be offered the test if their GP was not aware of their symptoms, or that the test existed. They were worried that the patients most confident to advocate for themselves, or those with proactive and well‐informed GPs, may be prioritised for screening, thereby placing responsibility on patients to report symptoms and request the test.

Two participants with busy work lives said they were currently taking PPI medication prescribed to friends and family. Consequently, their GP would never know the severity of their reflux. This also applied to those taking over‐the‐counter medication:

I would suggest there's a hell of a lot of people …who live on a diet of Gaviscon … and just make do. If they're not going to see a GP and they're not complaining about it… you'll never get to them.

(P9, M, intender, degree+)

Some felt swallowing the capsule would be their ‘job’ and felt pressure to perform their role in the test.

Getting it in my mouth and then not being able to swallow it or immediately puking it back out would be such a waste of money… I'd try very hard… I'm just not sure.

(P12, W, intender, degree+)

Others felt their ability to swallow the capsule would be contingent on the healthcare professional making them feel comfortable. Healthcare professional demeanour was deemed essential, and participants expected them to be adequately trained, build rapport, and be calm and reassuring.

Whilst the capsule sponge screening procedure relies on the user swallowing the capsule, participants were reassured that a healthcare professional would be present to ensure the procedure went to plan. They were also glad that a healthcare professional would be responsible for retrieving the sponge, as some felt they would be too ‘tentative’.

Nevertheless, healthcare professionals' involvement was problematic for some who anticipated problems with appointment availability and the timely communication of test results. Several participants described ‘real difficulty getting to see a GP’ and negative experiences in the NHS that made them doubt whether their GP would be able to effectively implement capsule sponge screening.

I think maybe I've just got this worry about the NHS and the doctors.

(P18, W, intender, degree+)

Despite this, some described the NHS as the 'envy of the world' and were confident that capsule sponge screening could be implemented effectively.

#### 3.2.5 Management of Barrett's

Whilst some felt that being diagnosed with Barrett's would help them to understand their reflux and might motivate behaviour change to reduce the risk of progression to oesophageal cancer, many said they would expect healthcare professionals to provide solutions to manage this risk. They believed healthcare professionals would treat and cure Barrett's, with some expecting them to ‘operate to remove the cells’ or provide medication, therefore placing the responsibility of care with clinicians rather than on the individual.

It might be a change in medication that deals directly with Barrett's… it may be something to target the inflammation…. And that's what I'd be looking for the medical professionals to advise me.

(P20, M, intender, <degree)

Most showed enthusiasm for endoscopic surveillance if diagnosed with Barrett's. They valued healthcare professionals' oversight, since the oesophagus is an internal organ, meaning patients cannot monitor themselves. Nevertheless, participants recognised their responsibility to chase, arrange and travel to surveillance appointments, making this a collaborative activity.

### 3.3 Coping With Uncertainty

Capsule sponge screening raised several uncertainties for participants to grapple with. This included the uncertainty of living with reflux, capsule sponge testing outcomes, and the test itself.

#### 3.3.1 Living With Reflux

Dealing with uncertainty was common amongst participants, who reflected on their lived experiences of reflux. Symptoms were reportedly unpredictable and inconsistent, and tried‐and‐tested management methods sometimes suddenly stopped working or varied in their efficacy.

I've got the omeprazole … but I don't find that really works very well. It's not super effective and in actual fact, sometimes that gives me the symptoms of reflux….

(P1, M, intender, <degree)

Participants described the process of weighing up their current lived experience of reflux (e.g., symptoms and their day‐to‐day impact) against a hypothetical future in which they might be diagnosed with Barrett's, making them at higher risk of cancer and leading to long‐term monitoring. Some were more concerned with the relative certainty of their present situation, that is, the concrete symptoms that they experience, rather than a potential future health problem such as Barrett's or oesophageal cancer. For these individuals understanding 'what is actually causing' their reflux and what can be done for it 'to be cleared' was more important than managing future risks.

Well, even before I knew about this Barrett's and if this wasn't happening, that would be, you know, where are all these pains coming from?

(P5, W, intender, <degree)

#### 3.3.2 Capsule Sponge Screening Procedure

The test procedure was viewed as another potential source of uncertainty. Many found it hard to imagine what having the test might be like due to the novel technique. Some drew comparisons with familiar experiences of swallowing tablets and endoscopy to navigate this procedural uncertainty. Hearing from others with experience of the test was important to some, to provide more certainty about what the test would be like.

Will it feel like pressure? Will it feel like pain? What would it feel like?W

(P26, M, intender, degree+)

Another consideration for some participants was the risk of something going wrong during the procedure. This possibility was overlooked by some, potentially due to healthcare professional involvement. Others were especially concerned by the risk of sponge detachment and felt it would defeat the point of the test if endoscopy was needed anyway (to retrieve the sponge). One participant said she was physically unable to undergo emergency endoscopy so did not think she could have the test in case something went wrong.

Well I'd decline it because I can't even turn my head to the left. And I'd wonder if it did go wrong how the heck they would then do an emergency endoscopy on me.

(P10, W, non‐intender, <degree)

Test accuracy was another source of uncertainty. Without sufficient accuracy, capsule sponge screening was seen as pointless, especially given the discomfort involved in the test. Nevertheless, some accepted that there is always a risk of something ‘slipping through the net because that's just life’. Participants seemed relatively accepting of false positives, where further testing resolves uncertainty.

But say for example you can eliminate 95% of your non‐Barrett's cases in one go then endoscopy as the next step for those people who are unlucky and show signs of Barrett's, yeah, then at that point. If it's a triage test, false positives don't worry me quite so much.

(P31, M, intender, degree+)

Contrastingly, participants seemed very averse to the idea of false negatives, potentially because this creates uncertainty about whether ‘something has been missed’, and for this reason, some preferred endoscopy to capsule sponge screening. With false positives, participants said the worst‐case scenario is that someone undergoes an endoscopy unnecessarily but would later get the ‘all clear’. Conversely, participants recognised that for false negatives, the worst‐case scenario is that Barrett's is missed, cancer develops and is only detected at a late stage; an outcome participants deemed as more severe.

#### 3.3.3 The Long‐Term Consequences of Capsule Sponge Screening

Participants grappled with the variety of outcomes that the test could have, including being given an ‘all‐clear’, receiving a ‘false‐positive’ or ‘false negative’ result, being diagnosed with Barrett's, needing surveillance but never developing cancer, and the chance of ultimately being diagnosed with cancer. Participants recognised that the more severe potential outcomes were also less likely, which was used to reassure them. Overall, most thought it was ‘better to be safe and sure’. For many, the small possibility of cancer seemed severe enough to warrant the almost certain discomfort of capsule sponge screening.

Contrastingly, some felt ‘ignorance is bliss’ and that the risk of developing cancer was so low that it was not worth undergoing screening, since the testing procedure would cause discomfort and anxiety whilst waiting for the results.

If it's a low risk even to have Barrett's… it's getting to the point where it's getting so minutely unlikely that you're kind of thinking to yourself well, you know, do I want to go through this.

(P36, W, non‐intender, degree+)

Some participants were disappointed to learn that Barrett's is often not actively treated and were keen for Barrett's to be removed, potentially to resolve uncertainty about whether or not it would progress to oesophageal cancer. Whilst most felt it was still useful to know if they had Barrett's even if it wouldn't be actively treated, some said they would not be interested in the test, if this were the case, since the diagnosis would lead to repeated endoscopies and could cause ongoing anxiety about the heightened risk of cancer associated with Barrett's.

What was the point in knowing I had Barrett's if there's nothing that can be done to prevent it and nothing that can be done to assist it and basically it's just a waiting game?

(P36, W, non‐intender, degree+)

Those with more complicated medical histories were more averse to the potential uncertainty that capsule sponge testing could introduce to their future. One participant described that his wife had recently undergone check‐ups for breast cancer recurrence and that this was a ‘nightmare’. Consequently, he wanted to avoid the stress of screening and monitoring and the state of limbo it would place him in.

Some participants said that as they aged, capsule sponge screening and Barrett's surveillance may no longer be a priority if their health or cognitive condition declined. Existing health issues were cited as a barrier to screening by one participant who said they already had enough to deal with. This individual seemed to prioritise dealing with a condition that they knew was actually a problem for them, rather than trying to prevent a condition that may or may not develop in the future.

I am going in for a prostate operation at the moment … I have other health problems that are more bothersome to me. … one thing I do psychologically is I deal with these other problems then I can cope with.

(P40, M, non‐intender, degree+)

#### 3.3.4 Confidence in Personal Health

The fact that Barrett's and oesophageal cancer lack distinct symptoms was another source of uncertainty, causing discomfort, especially for those who were unaware of this prior to the interviews. Some participants expected that their reflux symptoms might get worse, or that they might develop new symptoms such as dysphagia or oesophageal pain if they developed Barrett's or OAC. They described how they might use these symptoms to monitor their oesophageal health, meaning capsule sponge screening would be unnecessary. Many said that they felt healthy and did not think their symptoms warranted screening, but when they learned that these conditions often do not have symptoms until a late stage, this led them to question their oesophageal health, prompting some to reconsider screening.

Well because of what you said to me about it's symptomless. For peace of mind to myself, if it's somebody's opinion, a professional's opinion they advise me to have it, then I would have it.

(P38, M, non‐intender, <degree)

Conversely, where participants had recently attended an endoscopy, this provided certainty about their oesophageal health, leading them to think the capsule sponge test was unnecessary.

Death was described as an increasing certainty with age. Some were motivated to extend their life wherever possible, but others highlighted an essential trade‐off between enjoying and extending life. Most expected more health issues to arise as they aged.

We all want to live forever, although it seems that we're existing rather than living when we get to a certain age.

(P35, W, intender, <degree)

### 3.4 Proving Yourself as a Worthy Candidate for Capsule Sponge Screening

Many participants appeared to be assessing whether they were a worthy candidate for capsule sponge screening, in line with the Candidacy Framework [32]. They discussed their perceived eligibility for the test and described factors such as symptom severity and frequency, self‐efficacy to perform the test and how they would act on the test results.

Participants described experiencing severe symptoms that had often been ongoing for a long time or were difficult to control. These experiences fed into risk perceptions and views of candidacy. Language such as ‘I may well have Barrett's Syndrome’ reflects a desire to demonstrate their eligibility for the test. Others felt their symptoms were not severe or longstanding enough to warrant capsule sponge screening and therefore ruled themselves out as a candidate. This reasoning was often given by those less inclined to have the test, but appeared to be used to mask underlying procedural concerns. Some said capsule sponge screening would be good for those whose symptoms were more extreme but did not think it was personally relevant at present.

Not at the present because I don't think that I'm bad enough. If, in the future I felt that I had like a big problem, then I would. But not at the moment. No.

(P4, W, non‐intender, <degree)

Without being prompted, participants drew on general feelings of risk associated with age and family history to justify their (lack of) candidacy, demonstrating an understanding of other risk factors.

Luck and fatalism were also cited to justify test candidacy. Some felt they had always been unlucky in their health, so they were more likely to have Barrett's. Those who were more optimistic that their risk of Barrett's was low demonstrated less inclination for the test. Some believed they had little control over whether they would get cancer, making the test less valuable.

It's just basically your luck I think if you get cancer, isn't it? … You've just got to get on with it. If it turns into cancer, it turns into cancer. There's basically not a lot you can do about it.

(P32, M, intender, <degree)

Beyond this, many displayed ‘healthism’ tendencies [33] and overall efforts to maintain health were frequently described by participants. They felt capsule sponge screening was a good way to look after their health, which many believed was a personal responsibility and priority.

Many thought that ‘a lot of people have acid reflux’, with some saying, 'virtually all my friends are on omeprazole'. The normative nature of reflux seemed to diminish its perceived seriousness and also reinforce the view that capsule sponge screening would be highly sought after, meaning patients may need to justify why limited resources should be allocated to them. This seemed to be especially important given that many felt the NHS is currently under strain, meaning the availability of these tests may be limited.

For some, the idea of proving yourself as a worthy candidate for capsule screening was embedded in beliefs about self‐efficacy. Some were concerned that they may be unable to complete the test, either due to concerns about the string, swallowing the capsule, or gagging/vomiting whilst retrieving the sponge. Some felt it would be embarrassing to be unable to do it, and others said that they did not 'want to waste anybody's space or time in a queue for a test', especially if others were more worthy. Some were conflicted since they felt at risk of Barrett's but were concerned about their ability to perform the test, making them question their candidacy.

I don't know, could I bear the idea of this tickly thread down my throat and then have to wait 5 min and then have it trailed back up again… you might not be able to cope with it. You get it down and you can't cope with it, you get it pulled up then it's a waste of time. And it's a waste of resources, nurses, medication whatever.

(P36, W, non‐intender, degree+)

As noted above, one participant described being physically unable to undergo an endoscopy due to mobility issues. For her, there was no point in having capsule sponge screening if she would not be able to follow through with the subsequent stages.

Some also used altruistic reasoning to justify their candidacy, which many based on a ‘cost‐analysis’ to save the NHS money. Participants thought the test would be ‘a lot cheaper for the health service’ compared to treating oesophageal cancer. Using capsule sponge screening to triage those with reflux for endoscopy made sense to participants who said it ‘wouldn't be feasible to test everyone with acid reflux for Barrett's’ using endoscopy.

Some wanted a healthcare professional to confirm their candidacy, based on an assessment of their risk. This echoes earlier discussions regarding beliefs about who is responsible for reflux management and care,

If I fit into a category of that kind of person that is at risk … if a medical professional said that for peace of mind “test it”, then I would do.

(P38, M, non‐intender, <degree)

For some individuals, the possibility of cancer of the oesophagus had already been raised during endoscopy investigations, which seemed to reinforce their perception that they would be suitable candidates for the test.

Some also wanted to demonstrate that they would make capsule sponge screening worthwhile and reflected this when discussing the proactive actions they would take if they received a positive screening result. Some said that it would be important for them to act quickly to manage this situation. Participants discussed making lifestyle changes to better manage their reflux if diagnosed with Barrett's. Others were keen to ‘treat’ and ‘cure’ Barrett's, to remove its future threat.

Almost all participants said that they would definitely attend diagnostic endoscopy following a positive capsule sponge result, and some commented, ‘you'd be an absolute fool not to take it up’.

## 4 Discussion

### 4.1 Summary of Findings

This is the first in‐depth community‐based exploration of attitudes towards capsule sponge screening in people with reflux. Participants were enthusiastic about capsule sponge screening, but several considerations influenced their views. Beliefs about who is responsible for managing reflux varied widely. Uncertainty surrounding reflux‐related risks and the novel procedure caused discomfort for some, especially those seeking to minimise uncertainty about their health. Finally, views of candidacy varied based on participants' perceptions of risk, resource availability, self‐efficacy and the next steps after screening.

### 4.2 Integration With Existing Literature and Practice Implications

Consistent with the I‐SAM [15], we identified various capability, motivation and environmental‐based factors that seemed to influence capsule sponge screening intention. Low public awareness of the connection between reflux, oesophageal cancer and Barrett's was highlighted as a key consideration, as seen previously [34]. Interventions to increase awareness of the risks associated with long‐term reflux will be essential if capsule sponge screening is implemented.

Participants highlighted the identification of those eligible for capsule sponge screening and invitation procedures as key issues. Concerns around the need to self‐advocate for screening and remind healthcare professionals of their eligibility were evident. If introduced, capsule sponge screening will rely on the accurate recording of reflux symptoms to determine eligibility. Studies show that few individuals with reflux (~25%) consult their GP about it regularly [35]. This study highlighted how those with competing demands, including busy work lives or other health concerns, may not view their reflux as a priority to discuss with healthcare professionals, introducing potential inequities in access and uptake that have been well‐documented across cancer care pathways [36]. Challenges around the accurate recording of health information used to determine screening eligibility have been noted in lung screening contexts [37]. Interventions including electronic record reminders or embedding risk factor recording within NHS Health Checks have proven effective to document smoking behaviour [38, 39]. Similar interventions, alongside careful consideration of how inequalities in access might occur, will be essential to ensure capsule sponge screening reaches eligible populations. Alternatively, initiatives allowing patients to self‐refer, such as those already being trialled in this context [40, 41] and in breast screening for women over 70 [42] could be used to overcome issues around eligibility and screening invitation.

Evidence also suggests that healthcare professional communication can increase motivation and confidence to engage in screening, leading to more positive screening intentions [43]. Training for healthcare professionals to discuss reflux, related risks and screening will be essential alongside clear capsule sponge screening eligibility criteria and reliable measures to capture symptoms. Messaging campaigns encouraging those with persistent reflux to consult their GP will also be essential to ensure this test reaches its target audience.

Concerns around self‐efficacy to perform the test also seemed to act as a barrier for some, even if they were motivated to have it. Concerns about swallowing the capsule, the string, sponge retrieval and vomiting or gagging have been recorded elsewhere [22, 26, 28]. This study furthered our understanding by highlighting how the uncertainty around what the test would be like, and concerns about wasted resources explained hesitance to accept the test. Clear information materials describing the sensation of testing, preferably from first‐hand experience, should be developed to reassure participants and reduce procedural ambiguity where possible. Experience narratives have proven useful to increase engagement and self‐efficacy in cancer screening contexts [44] and could be an effective solution in this setting, provided that they provide a balanced description of capsule sponge screening to promote informed decision‐making [45]. These materials could also draw on comparisons with known sensations such as endoscopy and tablet swallowing, to help people create a mental model of the screening experience and dispel uncertainty and anxiety. Drawing on familiar experiences has been highlighted as a facilitator in other hypothetical screening contexts [46].

This study also highlighted uncertainties about what a Barrett's diagnosis would mean for patients. Similar uncertainties have been seen in real‐life studies of patients diagnosed with Barrett's, where a number of uncertainties around what Barrett's is, cancer risk, the role of surveillance, treatment, lifestyle and symptom management have been observed. Speculation about the future in those with Barrett's is common, and the ‘gap between what is and what might, could or even should be’ has been frequently reported by Barrett's patients [47, 48]. The fact that these imagined futures are potentially an issue amongst those with reflux, even before undergoing screening, highlights the pervasive nature of this phenomenon. Future research should consider how best to help patients cope with the variety of potential outcomes of capsule sponge screening, either through patient interventions, messaging, or through NHS pathways designed to support patients throughout their diagnosis and beyond [49].

Finally, views of candidacy (i.e., the perception that individuals are appropriate candidates for an intervention) seemed to influence capsule sponge screening decision‐making. The Candidacy Framework describes candidacy as a negotiated judgement about an individual's eligibility for healthcare, shaped by how people interpret their symptoms and health needs, as well as by healthcare systems and professionals that facilitate or constrain access to care [32]. This concept is particularly relevant to capsule sponge screening because eligibility is likely to be linked to the presence of reflux symptoms, which fluctuate over time and vary between individuals. Several aspects of this theory are applicable to this study's findings. The identification of candidacy was highlighted by participants, who used their symptoms, family history, age and general perceptions of health to justify their eligibility. Issues around navigation and the permeability of healthcare services were also highlighted, including concerns about appointment availability and resource allocation. The concepts of offers and resistance (i.e., the screening test offer and where a candidate decides against this course of action) were raised by those who did not want to undergo capsule sponge screening. In screening contexts, this theory has been used to understand individual, interpersonal and system‐level factors influencing uptake and driving inequities [50]. Accordingly, decisions about screening may be influenced not only by the intervention itself but also by whether individuals perceive themselves as legitimate candidates for it, and whether they are able to navigate the system to participate. Since this study highlighted limited awareness of the risks associated with reflux, interventions including decision support tools, public health campaigns and the provision of tailored risk information may help the public to identify their own candidacy and engage in capsule sponge screening [51].

As has been frequently documented elsewhere [52], some participants in this study demonstrated normative perceptions and perceived moral responsibility to undergo capsule sponge screening. These beliefs were couched in terms of reducing burden on healthcare services later down the line, a sense of moral responsibility to maintain good health or to appease healthcare professionals. Such beliefs inherently threaten informed decision‐making for cancer screening. The pervasive moralisation of screening and how to overcome this to promote informed decision‐making will be an important consideration when information materials for capsule sponge screening are developed.

### 4.3 Strengths, Limitations and Research Implications

This study purposively sampled men and women with differing intentions towards capsule sponge screening from a range of geographical and educational backgrounds, enabling us to capture diverse perspectives. Failure to recruit participants from ethnically diverse backgrounds is common in research settings [53, 54], and was reflected here in the fact that we did not recruit any participants from minoritised ethnic backgrounds. Ethnicity was not a specific sampling criterion, since reflux, Barrett's and OAC disproportionately affect White non‐Hispanic populations [55]. Instead, sampling focused on screening intention, geographical location and educational background. Nevertheless, individuals from ethnically minoritised backgrounds who experience reflux symptoms would still be eligible for screening and should be prioritised in future research. Our findings may not be fully transferable to ethnically diverse populations, particularly given evidence from wider cancer screening literature that has demonstrated how minoritised ethnic groups face distinct barriers to screening, including low health literacy [56], cancer stigma [57] and reduced access to primary care [58].

Our purposive sampling of non‐intenders is a strength, since typically these individuals are less likely to engage in research, but their views are essential to understand potential uptake, inequalities in access and informed decision‐making. This group's views are seldom captured, and non‐intenders' attitudes to capsule sponge screening have not been explored in depth before. Our community‐based recruitment was a strength compared to previous studies that have recruited via trials or market research panels. However, it may still have introduced bias, for example, if people attending community groups were more socially connected or wealthier than the general population [59].

By using qualitative methods, we were able to gain an in‐depth and nuanced understanding of barriers and enablers to capsule sponge screening across a broad range of individuals. Nevertheless, qualitative studies lack generalisability. In particular, research studies are especially susceptible to healthy volunteer bias, and research volunteers are more likely to engage in screening [60, 61]. Many of our participants had previously consulted their GP about their reflux, yet we know that reflux‐related consultation rates are fairly low [35]. Further quantitative studies should be conducted to consider how often issues identified here occur on a population level.

Another limitation of this study is its hypothetical nature. The field of oesophageal cancer screening and early detection is constantly evolving. The recent BOSS trial [62] raised particular questions about the value of identifying and monitoring Barrett's, which makes it difficult to predict what the future of potential Barrett's screening will look like. Whilst it can be viewed as beneficial to research attitudes to novel screening interventions prior to their introduction so that findings can influence implementation, it can also make it difficult to provide participants with concrete information. For example, during the interviews, participants were told that the test would likely be introduced in primary care settings, but these tests can be delivered in other ways, such as in mobile units [34], secondary care [28] and community settings [40]. In our study, some participants commented that the GP surgery was a preferable location to have the test, given its familiarity and convenience, but, like in previous studies [28], some said they would feel safer in a secondary care setting.

Whilst data collection was ongoing, a new pilot investigating the feasibility of capsule sponge screening in pharmacies was announced [63]. This demonstrates the constantly shifting landscape of hypothetical research, which could limit the applicability of findings if the context presented does not align with the way an intervention is actually implemented. Furthermore, asking participants to imagine how they would behave may not reflect their actual decision‐making, which will likely depend on personal circumstances at the time, as has been well‐documented in literature on the intention‐behaviour gap [64]. As such, further work exploring barriers and enablers to capsule sponge screening, if and when it is implemented, would be recommended.

## 5 Conclusions

This is the first study to explore public attitudes to capsule sponge screening with a specific focus on those less positively inclined towards the test and more diverse community samples. Whilst most were enthusiastic about the test, queries about who was responsible for reflux care and who was eligible for the test were common. Uncertainty was a widely reported source of discomfort. Raising awareness of reflux and related risks to support informed decision‐making will be key, and future research should consider barriers, enablers and equity in the context of real‐life implementation.

## Author Contributions

**Ninian Schmeising‐Barnes:** conceptualisation, methodology, formal analysis, data curation, writing – original draft, project administration. **Emma Lidington:** conceptualisation, methodology, formal analysis, writing – review and editing, supervision, funding acquisition. **Elisavet Moschopoulou:** validation, formal analysis, writing – review and editing, supervision. **Daksha Trivedi:** methodology, writing – review and editing. **Jo Waller:** conceptualisation, methodology, validation, writing – review and editing, formal analysis, supervision, funding acquisition.

## Ethics Statement

This study was approved by the Queen Mary University of London Research Ethics Committee (QME24.0465).

## Consent

All participants gave their informed consent before participation in this study.

## Conflicts of Interest

The authors declare no conflicts of interest.

## Supporting information


Supporting File

## Data Availability

The full dataset is available upon request.
