# Supplementary material for: Barriers and Enablers to Capsule Sponge Screening Among People With Heartburn: A Community‐Based Qualitative Study in the UK
Source: Health Expect. 2026 Oct 15;29(5):e70919. doi: 10.1111/hex.70919 (PMC13670410; doi:10.1111/hex.70919)
Supplement: Supplementary file 1 [file HEX-29-e70919-s001.docx]

**Supplementary materials:**

**Supplementary material 1: COREQ checklist**

**COREQ (COnsolidated criteria for REporting Qualitative research) Checklist**

A checklist of items that should be included in reports of qualitative research. You must report the page number in your manuscript where you consider each of the items listed in this checklist. If you have not included this information, either revise your manuscript accordingly before submitting or note N/A.

| **Topic** | **Item No.** | **Guide Questions/Description** | **Reported on Page No.** |
| --- | --- | --- | --- |
| **Domain 1: Research team and reflexivity** |  |  |  |
| *Personal characteristics* |  |  |  |
| Interviewer/facilitator | 1 | Which author/s conducted the interview or focus group? | 4 |
| Credentials | 2 | What were the researcher’s credentials? E.g. PhD, MD | 5 |
| Occupation | 3 | What was their occupation at the time of the study? | 5 |
| Gender | 4 | Was the researcher male or female? | 5 |
| Experience and training | 5 | What experience or training did the researcher have? | 5 |
| *Relationship with participants* |  |  |  |
| Relationship established | 6 | Was a relationship established prior to study commencement? | 4-5 |
| Participant knowledge of the interviewer | 7 | What did the participants know about the researcher? e.g. personal goals, reasons for doing the research | 4 |
| Interviewer characteristics | 8 | What characteristics were reported about the inter viewer/facilitator? e.g. Bias, assumptions, reasons and interests in the research topic | 5 |
| **Domain 2: Study design** |  |  |  |
| *Theoretical framework* |  |  |  |
| Methodological orientation and Theory | 9 | What methodological orientation was stated to underpin the study? e.g.  grounded theory, discourse analysis, ethnography, phenomenology, content analysis | 4 |
| *Participant selection* |  |  |  |
| Sampling | 10 | How were participants selected? e.g. purposive, convenience, consecutive, snowball | 3 |
| Method of approach | 11 | How were participants approached? e.g. face-to-face, telephone, mail, email | 3-4 |
| Sample size | 12 | How many participants were in the study? | 5 |
| Non-participation | 13 | How many people refused to participate or dropped out? Reasons? | 5 |
| *Setting* |  |  |  |
| **Topic** | **Item No.** | **Guide Questions/Description** | **Reported on Page No.** |
| Setting of data collection | 14 | Where was the data collected? e.g. home, clinic, workplace | 4 |
| Presence of nonparticipants | 15 | Was anyone else present besides the participants and researchers? | - |
| Description of sample | 16 | What are the important characteristics of the sample? e.g. demographic data, date | 3, 5-6 |
| *Data collection* |  |  |  |
| Interview guide | 17 | Were questions, prompts, guides provided by the authors? Was it pilot tested? | 4 |
| Repeat interviews | 18 | Were repeat inter views carried out? If yes, how many? | N/A |
| Audio/visual recording | 19 | Did the research use audio or visual recording to collect the data? | 4 |
| Field notes | 20 | Were field notes made during and/or after the interview or focus group? | 5 |
| Duration | 21 | What was the duration of the inter views or focus group? | 5 |
| Data saturation | 22 | Was data saturation discussed? | N/A |
| Transcripts returned | 23 | Were transcripts returned to participants for comment and/or correction? | 4 |
| **Domain 3: analysis and findings** | | |  |
| *Data analysis* |  |  |  |
| Number of data coders | 24 | How many data coders coded the data? | 4 |
| Description of the coding tree | 25 | Did authors provide a description of the coding tree? | 4 |
| Derivation of themes | 26 | Were themes identified in advance or derived from the data? | 4 |
| Software | 27 | What software, if applicable, was used to manage the data? | 4 |
| Participant checking | 28 | Did participants provide feedback on the findings? | N/A |
| *Reporting* |  |  |  |
| Quotations presented | 29 | Were participant quotations presented to illustrate the themes/findings?  Was each quotation identified? e.g. participant number | 7-14 |
| Data and findings consistent | 30 | Was there consistency between the data presented and the findings? | 7-14 |
| Clarity of major themes | 31 | Were major themes clearly presented in the findings? | 7-14 |
| Clarity of minor themes | 32 | Is there a description of diverse cases or discussion of minor themes? | 7-14 |

Developed from: Tong A, Sainsbury P, Craig J. Consolidated criteria for reporting qualitative research (COREQ): a 32-item checklist for interviews and focus groups. *International Journal for Quality in Health Care*. 2007. Volume 19, Number 6: pp. 349 – 357

**Once you have completed this checklist, please save a copy and upload it as part of your submission. DO NOT** **include this checklist as part of the main manuscript document. It must be uploaded as a separate file.**

**Supplementary material 2: Pre-screener questionnaire**

**PANORAMA: Public attitudes to capsule sponge test screening for Barrett’s Oesophagus among people living with acid reflux.**

*Thanks for your interest in our research study. We are interviewing people with acid reflux to find out their thoughts on a new test that might be offered in future.*

*People with long-term acid reflux are at a slightly higher risk of developing a pre-cancerous condition known as Barrett’s Oesophagus. A new test has been developed to detect Barrett’s Oesophagus, known as a capsule sponge test.*

*We would like to hear about your experiences with acid reflux and your views on capsule sponge test screening. More information will be provided about the test before the interview.*

*For more information on what the study involves, please visit: <<<link to PIS>>>.*

**To sign up, please complete the following questions. Unfortunately, we are not able to interview everyone for this study. We will use this information to invite people with a range of experiences and backgrounds.**

**We will store your responses to this form for up to 1 year.**

*If you are happy for us to store your responses for up to 1 year, please select “Yes” below:*

- Yes
- No <<<skip to ineligible section>>>

***What is your age?*** [free text, validation: integer 1-100]

_____________

**Which of the following best describes your gender?**

- Man
- Woman
- Non-binary
- Prefer to self-describe (specify, if you wish)

If Gender=Male and Age is NOT=55-79 OR If Sex=Female and Age is NOT=65-79 <<skip to ineligible section>>

**Acid reflux medication use**

***Over the last 6 months, have you taken medication for heartburn, indigestion or acid reflux at least twice a week? This could be over-the-counter medicine like Gaviscon or Rennie, or a prescribed medicine like Omeprazole or Lansoprazole.*** [single choice radio buttons, forced response]

- Yes
- No <<skip to ineligible section>>
- Unsure <<skip to ineligible section>>

***Have you ever been diagnosed with oesophageal cancer (cancer of the gullet or food pipe)?*** [single choice radio buttons, forced response]

- Yes<<skip to ineligible section>>
- No
- Prefer not to say <<skip to ineligible section>>

***Have you ever been diagnosed with Barrett’s Oesophagus*** [***<<link to definition>>***](https://www.cancerresearchuk.org/about-cancer/other-conditions/barretts-oesophagus)***?*** [single choice radio buttons, forced response]

- Yes <<skip to ineligible section>>
- No
- Prefer not to say <<skip to ineligible section>>

***[HIDDEN UNTIL BEST4 SCREENING TRIAL STARTS] Have you ever heard of the BEST4 screening trial?*** [single choice radio buttons, forced response]

- No, I have never heard of it
- Yes, but I have not been invited to take part in the trial
- Yes, I was invited to take part in the trial but decided not to
- Yes, I am taking part in the trial <<skip to ineligible section>>
- Prefer not to say <<skip to ineligible section>>

***Have you ever had a capsule sponge test (also known as ‘cytosponge’)?*** This involves swallowing a capsule on a string to pick up problems with the oesophagus [single choice radio buttons, forced response]

- Yes <<skip to ineligible section>>
- No
- Not sure
- Prefer not to say

***Which UK region do you live in?*** [single choice radio buttons]

- North East
- North West
- Yorkshire and the Humber
- East Midlands
- West Midlands
- East of England
- London
- South East
- South West
- Wales
- Scotland
- Northern Ireland
- I currently live outside of the UK <<skip to ineligible section>>

**What is the highest level of education you have completed?**

*If you received education outside the UK, please select the closest equivalent.*

- Secondary school (up to 16 years), or below
- Higher or secondary or further education (A-levels, BTEC, etc.)
- Degree level or above

***Intention to have CST screening***

*You can watch this video to see what this test looks like:*

*This test involves swallowing a small capsule roughly the size of a multi-vitamin pill. The capsule contains a small sponge with a string attached to it. When you swallow it with water, the capsule dissolves and the sponge expands in your stomach. The string is then pulled to retrieve the sponge. As the sponge comes back up, it collects cells from the entire length of your oesophagus. These cells can be tested to see if you have Barrett’s Oesophagus.*

If you were invited to have the capsule sponge test locally, would you take up the offer? [single choice radio buttons, forced response]

- Yes, definitely
- Yes, probably
- No, probably not
- No, definitely not
- Don’t know

***Please indicate your preferred method for the interview:***

(Please note, if you would like to complete the interview in-person, we can cover up to £50 of travel costs. We can only offer in-person interviews in certain locations, but we will do our best to offer the interview in your preferred method.)

- Telephone
- In-person
- Video-call (Microsoft Teams)

***What is the best way to contact you?***

- Email
- Telephone
- Letter

**Contact details**

Name: ____________________________ (free text)

Email address: _____________________________ (free text)

Phone number: _____________________________ (free text)

Home Address: _____________________________ (free text)

City: ________________ (free text)

Postcode: ______________ (free text)

**Ineligible section text:**

Unfortunately, you do not meet the requirements for this study. Thank you for taking the time to complete this survey. If you’re interested in learning about our other research studies, you can visit: [REDACTED]

**Thank you section:**

Thank you for completing this form and for showing your interest in an interview.

If you have been selected, we will be in contact with you within 28 days.

If you do not hear from us this means that you have not been selected for an interview at this time. We will hold onto your details for 1 year in case more interview slots become available. If you no longer want to take part in an interview you can let us know by emailing: [[XXXXX]](mailto:n.schmeising-barnes@qmul.ac.uk).

**Supplementary material 3: Topic guide**

1. **REFLUX:** Can you start off by telling me all about your reflux - like what symptoms you get, when it started, how you manage it and so on
   a. Feelings about reflux, worry about impact on future health, what others think
2. **BARRETT’S:** Have you ever heard of a condition called Barrett’s Oesophagus?
   a. If yes: can you tell me what you’ve heard?
   b. If no: give blurb about Barrett’s

- First thoughts about info about Barrett’s (link with reflux/ feelings? makes sense? feelings about monitoring? Personal perceived risk?)

3. **GENERAL SCREENING EXPERIENCE:**

- Previous attendance, why/why not attended

*(read different versions of blurb depending on whether they watched video) + ADDITIONAL INFO*

4. **ATTITUDES TOWARDS CSTS:**

- How do you feel hearing this info?
- Information needs (type, source, seeking additional info, support from others?)
- What might affect your decision (able to do prep? Worries about the procedure? need for test, motivation to have the test, benefits of finding Barrett’s, location, process [no anaesthetic/ nurse])
- Feelings when invited to have CSTS
- General intention
- Anything that might change mind?
- How you would feel if you had a positive / negative result

5. **NEED FOR DIAGNOSTIC TESTING (ENDOSCOPY)** – anything that would stop you going, previous experience, wait time, getting an appointment
6. **THOUGHTS/ FEELINGS TOWARDS DIAGNOSIS OF BARRETT’S** (monitoring/NHS/ cancer worry/behaviour/hypervigilance)
7. **FEELINGS TOWARDS REFLUX:**

Does hearing info on Barrett’s / OAC change way look at reflux? (behaviour change, risk of cancer)

**Supplementary material 4: Capsule sponge test information video:**

[**https://www.youtube.com/watch?v=s7X9z6qlNUI**](https://www.youtube.com/watch?v=s7X9z6qlNUI)

**Supplementary material 5: Thematic framework analysis codebook:**

| 01 UNDERSTANDING OF THE TEST | Views surrounding their understanding of the test, why it is important and how it works. |
| --- | --- |
| 01.1 Purpose | What the test is looking for, views on why this is important |
| 01.2 Personal relevance | Whether they feel this test is important to them, or whether it might be useful for others, reason why they feel it is relevant/not for them |
| 01.2.1 Risks associated with reflux | Views around the general risks associated with acid reflux |
| 01.2.1.1 Awareness of risks | General risks associated with reflux (excluding OAC and Barrett's) |
| 01.2.1.1.1 Awareness of Barrett's | Reported knowledge of Barrett's, how they might feel about their level of knowledge, misconceptions about Barrett's |
| 01.2.1.1.2 Awareness of link with cancer | Knowledge of the link between OAC and reflux, misconceptions, lack of knowledge- changed to include awareness of link to other cancers (typically stomach and throat cancer) as this can be classified as a misconception |
| 01.2.1.1.3 Information sources for risk | Who/where people get information about the risks associated with reflux (Inc. Barrett's/OAC/other risks) - their thoughts on this information. |
| 01.2.1.2 Perceived personal risk of Barrett's or OAC | Individuals' views on how likely Barrett's / OAC are for them |
| 01.2.1.2.1 Other factors influencing risk | Factors influencing people’s perceived personal risk (including factors that both increase and reduce their risk. Also includes awareness of risk factors, even if not contextualised into own risk perceptions |
| 01.2.1.3 Emotional responses to risk | How participants feel about their risk, for example - worry (or lack of) |
| 01.2.2 Symptoms | Comments relating to participants’ reflux symptom |
| 01.2.2.1 Nature of symptoms | Types of symptoms people are experiencing, how often they get them and how severe they are. |
| 01.2.2.2 Onset and Duration | When symptoms started and how long they've been going on for |
| 01.2.2.3 Causes | Things causing the reflux or making it worse |
| 01.2.2.4 Impact of symptoms | How they think the symptoms affect their day to day life |
| 01.2.3 MANAGEMENT | Comments relating to the ways in which participants manage their reflux and how they find this |
| 01.2.3.1 Medication | General information about what type of medication people take to manage their reflux |
| 01.2.3.1.1 Effectiveness | Views on how well the medicine works and why they might think it is effective or not. |
| 01.2.3.1.2 Adherence | Reports on how well participants adhere to medication and factors that make it easier/more difficult to follow recommended treatment schedules. |
| 01.2.3.1.3 Concerns | Concerns about reflux medications, sources of information, emotional responses to these concerns and how to resolve concerns. |
| 01.2.3.1.4 Initiation | When medication was started, why they decided to start medication and how they went about initiation. |
| 01.2.3.1.5 Medication being used | Names of medication being used. |
| 01.2.3.2 Alternative approaches to management | Other ways (besides medication) that people manage their reflux, and motivation for why they do this, how they find alternative approaches to management reflux |
| 01.2.3.3 Reasons for managing reflux | Why people want to manage their reflux (or why they don't manage their reflux) either by medication or alternative methods. |
| 01.2.3.4 help seeking | Decisions to seek help relating to their reflux, who they go to, when and why |
| 01.2.4 Awareness of Reflux as a health condition | Whether participants knew that reflux is a problem that can cause serious health threats, or whether they think of it as a more transient and lifestyle related condition. |
| 01.2.5 Salience of reflux and related conditions in wider context of health | Compare to other conditions, how important is their reflux, is it the most important condition or do they have lots of other health conditions that they deem are more important? |
| 01.3 Effectiveness | Whether the test is likely to reduce cancer, whether it can actually detect Barrett's, views on comparable effectiveness to endoscopy, views on False Positives |
| 01.3.1 Cancer beliefs | Any other cancer beliefs not covered in subcodes |
| 01.3.1.1 Severity | Beliefs that cancer is severe/ specific beliefs about oesophageal cancer and how severe that it, any insights into knowledge of the severity of OAC, or lack off |
| 01.3.1.2 Screening | Any other screening beliefs not covered in subcodes |
| 01.3.1.2.1 previous experiences | Previous experiences of screening in national screening programmes such as breast, bowel, colorectal and prostate screening. How they found it. |
| 01.3.1.2.2 reasons for & against screening | Participants view on why screening is good or bad. Things that they link about it and things that they see at negatives about screening |
| 01.3.1.2.3 Awareness of screening as a concept | Whether participants really understood what screening is e.g. asymptomatic nature, whether they understood the question when asked about their previous screening behaviour or if they cited other health checks as they thought this was what screening is. |
| 01.3.1.3 Treatment | Views on efficacy of cancer treatment, what it is like, whether they would be interested in having it and why it is important, and especially linked into the participants motivation to have test |
| 01.3.1.4 Prevention | Beliefs around how one can prevent cancer and how they can prevent it. |
| 01.3.1.5 Symptoms | Participant beliefs around the symptoms of cancer and how they can spot cancer. |
| 01.3.1.6 Early detection | Participant discusses early cancer detection including benefits or negatives of early cancer detection. |
| 01.3.1.7 emotional responses to cancer | Discussion of emotions in relation to cancer e.g. fear, worry etc. |
| 01.3.8 causes | Participant views surrounding the causes of cancer e.g. genetics, lifestyle, luck, fate etc. |
| 01.3.9 other beliefs | Other beliefs relating to cancer. |
| 01.3.2 Perceived importance of endoscopy follow up | Views about how important endoscopy (either for monitoring or diagnosis) is and why it is or isn't important |
| 01.4 INFORMATION NEEDS | Information needs for capsule sponge test screening |
| 01.4.1 Risks associated with reflux | Information about the link between reflux, Barrett's and OAC - likelihood of progression, risk factors etc |
| 01.4.2 Information about the test | Features about the test, what the risks are, mechanisms of the test, where is would be done, why this information is important |
| 01.4.3 What happens next | Information about what happens after CSTS - results delivery, what happens if the test is positive, what happens if you're diagnosed with Barrett's, prevention of OAC. Why this information is important |
| 01.4.4 capsule sponge test screening in action | Views around seeing the capsule sponge, seeing someone else have a capsule sponge test, hearing real life accounts of others who have had the test, and how this helps/hinders perceptions of the test |
| 01.4.5 Sources of information | Where participants expect to hear about capsule sponge test screening, where they might look for information and how they feel about different sources of information |
| 01.5 Attributes of the test | Features of the test that participants comment on e.g. the speed of the test, cost, invasiveness. |
| 02 INTENTIONS | Intentions to have the test or not |
| 03. Physical ability to perform the test | Comments surrounding self-efficacy to perform the test procedure and factors enabling and acting as barriers to the act of undergoing testing. |
| 03.1 Swallowing | Barriers around swallowing the tablet, and/or enablers to swallowing the tablet |
| 03.2 String | Barriers and enablers to CSTS surrounding the string and how they influence intention |
| 03.3 Retrieving sponge | Barriers and enablers to capsule sponge screening resolving around retrieving the sponge |
| 03.4 previous experiences of other medical procedures | Participants' account of previous experience of having had endoscopy and how this influences their views on having CSTS and endoscopy for follow up and monitoring following Barrett's diagnosis |
| 03.4.1 hearing about other's experiences of procedures | Participants perceptions about how hearing about someone else’s experiences of CSTS would affect how they feel about the test or approach the test overall |
| 03.5 Endoscopy Capability | Views on how able participants are likely to be able to have endoscopy, and factors influencing individuals perceived ability to have endoscopy (either for monitoring or diagnosis |
| 03.6 Anticipated discomfort | How uncomfortable they think the test would be, views on pain. |
| 03.7 Other factors influencing physical ability to perform test | Factors influencing self-efficacy to perform the test, not listed above. |
| 04 Emotional responses around CSTS | Participant’s descriptions of the emotional reactions CSTS stimulated within them |
| 04.1 Emotional responses | Psychological capability enablers and barriers to CSTS |
| 04.1.1 Fear | Fear of endoscopy, reasons for fear, impact on views towards endoscopy (for monitoring and for diagnosis) |
| 04.2 emotional experiences or reactions associated with endoscopy and other procedures | Participants' account of previous experience of having had endoscopy and how this influences their views on having CSTS and endoscopy for follow up and monitoring following Barrett's diagnosis |
| 05. ENVIRONMENTAL FACTORS INFLUENCING CSTS | Other environmental factors influencing CSTS views and intentions |
| 05.1 Social influences | Social comparison, others’ views on CSTS and how it influences their views, endorsement, who by and why this is important |
| 05.2 Healthcare system factors | Factors relating to the healthcare systems that influence ability or desire to perform the test, e.g. NHS waiting lists, strain, appointment availability etc. |
| 05.3 Travel and transport | Considerations relating to travel and transport for CSTS |
| 05.4 Who offers the test-setting | Comments around the setting the test should be offered in and the healthcare professional that should be offering it, e.g. training and rapport building. |
| 05.5 Other logistical factors | views on how the test should be offered - where, who by, when the test will be offered - 5 Ws |
| 06 Wanting to know | Participants reflections on whether they would want to know if they had Barrett’s or not and how they would utilise the information if they did find out. |
| 06.1 Treatment | Views about wanting to know about a condition so that you can treat it whether this be Barrett's or OAC |
| 06.2 Prevention | Wanting to know about Barrett's so that you can prevent progression to OAC |
| 06.3 Reassurance | Wanting to know that you don't have Barrett's to provide reassurance, or wanting to know you've got Barrett's so that you are reassured that you are being monitored in case anything does develop |
| 06.4 Monitoring | Views around wanting to have CSTS so that they can be monitored if Barrett’s is diagnosed |
| 06.5 Wanting to fix the problem | Views around wanting to have CSTS so they can understand their reflux better, potentially leading to better symptoms control and fewer day to day effects of reflux on their lives. |
| 06.6 Other reasons for wanting to know | Other reasons for wanting to know |
| 07 General approaches to health | Participants general attitudes towards their health and the general approach they take in managing their health |
| 07.1 Previous healthcare experiences | Participants recount previous healthcare encounters in primary and secondary care and reflected on how these experiences may influence the way that they feel about or approach CSTS. |
| 08 Other | Other comments not covered elsewhere in the coding framework but deemed important. |

**Supplementary material 6: Reflexivity statement**

Reflexivity is defined as “a set of continuous, collaborative, and multifaceted practices through which researchers self-consciously critique, appraise, and evaluate how their subjectivity and context influence the research processes.” Reflexivity is considered an important way for qualitive researchers to improve rigor and the quality of work and requires researchers to consider their position within the research and how their experiences, beliefs and biases impact the research.

Throughout the research process, the authors reflected upon their position within the research and the impact this had. Whilst all authors contributed to the data collection phase, [author1initials] led the analysis and derived themes from the data. This statement summarises reflections [author1initials] noted throughout the data collection, analysis and write up of this work and provides suggestions on how these might have impacted upon the findings.

**Personal characteristics:**

[author1initials] is a female, cisgender, white-British PhD student. [author1initials] was 26 years old at the start of data collection and had been working in the field of cancer behavioural science for approximately 3 years. [author1initials] has a MSc in Health Psychology and has a close family history of cancer.

[author1initials] suggested that these characteristics might have impacted this research in a number of ways. [author1initials] had previously attended cancer screening herself and these experiences are likely to have shaped her perceptions of cancer screening.

[author1initials] worked in the NHS in the past. She reflected that this employment history had given her firsthand experience of the strain that the NHS is under and instilled a certain allegiance to the NHS. She acknowledged that she sometimes felt defensive when individuals were critical of the NHS and also felt a personal sense of pride when participants shared positive thoughts of the NHS. It is feasible these personal ties to the NHS impacted how she interpreted the data and her interactions with participants during data collection.

Having worked in the field of cancer screening for some time at the time of data collection, [author1initials] reflected that she is highly knowledgeable on this topic. On one hand, she was sometimes shocked at participants limited awareness of cancer screening, but also expressed surprise when individuals demonstrated a greater understanding than expected. She reflected that she found it difficult to resist the righting reflex when participants expressed misconceptions surrounding cancer screening.

[author1initials] noted that she is quite health conscious and tends to be anxious about her health and that of others. She also noted that before working in the field of cancer screening, she was fearful of cancer but working in the field has made her more aware of the treatment and survivability. She considers herself as “someone that would rather know about cancer” and therefore found it difficult to relate to those who said they’d rather not know about cancer, and those that would refuse treatment.  During the groups she reflected on the fact that some individuals were less concerned about their health or disease, especially with age, or seemed not to be worried about a potential cancer diagnosis. This conflicted with her personal views, which she put down to her younger age and personal family history of cancer. She noted that this dissonance might have influenced how she interacted within the focus groups and/or analysed the data.

[author1initials] also noted that she has never particularly experienced poor health so found it hard to empathise with participants who said they struggled to have adequate access to healthcare e.g. due to limitations in NHS provisions.

[author1initials] is generally holds positive views about cancer screening, although reflected that her views have changed since she started working in the field. Where she was previously very pro screening, she now considers herself to be more ‘pro informed choice’. She was initially shocked by how positive most individuals were about screening, sometimes even without any justification, but also remembered that held these views until recently. She also recognised that she still felt internally conflicted when participants expressed negative views towards screening and suggested this might have impacted how she interpreted the data or interacted with participants.

**Fair dealings:**

Throughout the interviews and analysis, the researchers aimed to instil the principle of fair dealings. According to Dingwall, ‘fair dealings’ is a way of reducing bias within qualitative research by incorporating a wide range of views. When reporting the findings, [author1initials] reported a range of views expressed within the groups including those less commonly reported. However, a range of factors might have impacted the extent to which this aim was achieved. Firstly, [author1initials] spoke to friends and family about capsule sponge screening before and around the groups, which could have influenced her perception of the ‘norm’ views. Secondly, [author1initials] reflected on the fact that she was motivated to uncover a range of views within the focus groups. She was aware of the fact that positive views about capsule sponge screening were more prominent and as such felt she might have probed for negative views more than appropriate to increase the amount of data available about these negative views.

[author1initials] views herself as a confident and chatty individual and suggested she was frustrated when some individuals didn’t participate fully. She also reflected that she sometimes avoided asking individuals further probing questions if she was worried they might go off on a tangent, which might have impacted the data collection.

**Power:**

Some participants completed the interviews virtually from their homes, or with [author1initials] visiting their homes, meaning as the researchers we had a small insight into participants’ private lives. In other instances, the participants came to the campus. This led to feelings of hosting and a slight amount of performance on [author1initials] part, to ensure their comfort.

During the data collection phase, participants might have viewed the researchers as in a position of power over them despite efforts to avoid this. [author1initials] was especially conscious of this, and as such it is feasible she overcompensated for this meaning the interactions were less natural throughout the interviews.

**Team data analysis:**

Whilst [author1initials] was primarily responsible for coding and data analysis, she met regularly with [author5initials] and [author2initials] to discuss the emerging themes, throughout data collection and analysis. [author5initials] and [author2initials] both have extensive qualitative experiences and provided unique perspectives. They suggested ideas for themes and highlighted aspects of the data that they found personally interesting. Both [author5initials] and [author2initials] are more experienced in qualitative methods that myself ([author1initals]), so there were definitely instances where I felt I should give their ideas more space within my analysis. Their comments and thoughts undoubtedly impacted the way I asked interviewing and analysis, but I recall making a particular effort to embrace the subjectivity of the process and tried to remain true to my own perspectives of the data where possible.

**Reliability of coding:**

At the start of analysis, [author5initials] and [author4initals] reviewed the coding for three transcripts. This exercise did not result in any changes to the coding framework but was used to check that I ([author1initials]) was coding the transcripts sufficiently and to ensure the coding framework functioned well within the data and could be used easily.

**Comparison of data within and across the dataset:**

A matrix was used to compare between and within cases via the framework approach. I compared within cases to get an idea of how perceptions changed within interviews with increasing awareness and knowledge gained. Comparisons across cases were drawn to identify themes and exceptions to these rules to develop and strengthen the stories told within the themes.

**Use of software for analysis:**

NVivo was used to facilitate coding and analysis. I used annotations with NVivo to write reminders of topics to explore, potential latent interpretations and topics to check between participants. I also used NVivo and mind mapping to draw comparisons between descriptive themes that were developed during earlier stages of analysis and create latent, more interpretative themes.

**Supplementary material 7: Supplementary quotes**

Theme 1: A collaborative affair: negotiating responsibility for reflux management and care

| Subtheme | Concept | Support quote(s) [participant characteristics] |
| --- | --- | --- |
| *Reflux risk awareness* | Most had never heard of Barrett’s | “I mean yeah, I’d never heard of Barrett’s before speaking to you so it's new to me” [P6, W, intender, degree+]  “I didn’t honestly think of any kind of risks. I’d never heard of any risks so I don’t know, I’ve never read anything up about it so I didn’t know there was risks to it.” [P27, W, intender, degree+] |
|  | Unawareness of the link between cancer and OAC | “No, I didn’t realise it would lead to throat cancer or cancer of the gullet. I didn’t realise that it would lead to that.” [P32, M, intender, <degree]  “I was a bit shocked, to be honest. Yes. I had no idea and I- I think it- Indigestion, you tend to sort of pass off as something *oh perhaps you have overindulged* or something like that, and it’s- It’s one of those- *I’ve got a bit of a headache,* or whatever, and you just deal with it. But actually, the implications could be a lot more serious, and- You know. So no, I had no idea of that sort of connection.” [P17, W, non-intender, degree+] |
|  | Confidence in ability to assess personal risk after extensive research | “Yeah, yeah I’ve- Well it was a long time ago now I investigated this, I know there are some, cancer of the oesophagus and other stuff… [when asked whether he feels at risk] … Yes, but maybe 5% more than the rest of the population, I don’t feel the crosshairs are on my forehead or anything.” [P11, M, intender, degree+] |
|  | Healthcare professionals cannot share information about reflux risks if patients do not consult | “If they’re not going to see a GP and they’re not complaining about it and they’re just literally pumping more and more Gaviscon or the equivalent- milk and magnesium, it would have been in olden times. There’s no way- you’ll never get to them. I mean, people who are actually booked up with that Gaviscon, omeprazole or lansoprazole or something, you kind of know off your medication charts- you’ve got them.” [P9, M, intender, degree+]  “The doctor should really talk to them but I’ve never even been asked to go and speak to the doctor about my reflux which- That’s why I’m saying, you know, what way will the testing- How does the testing work because as I say, I’ve seen my asthma nurse, she arranged for the script, I’ve just gone on to repeat scripts.” [P36, W, non-intender, degree+] |
| *Capsule sponge screening eligibility and procedure* | Healthcare professionals invertedly gatekeeping the test | “How widely known is this test then? Because I knew nothing about it, you know, the doctor has never said anything. And the other thing, I suppose, I’m just thinking as you’re saying this, this all is, you know, is very well, but we have- I don't know what it’s like where you are, in [COUNTRY] we have real difficulty getting to see a GP or to talk to a GP. So if this test was available, how would we know about it?” [P23, W, intender, <degree]  “I suspect it’s underplayed by GPs. They’re more concerned in giving you omeprazole and lifestyle advice but they’re not necessarily concerned with managing what may or may not be a small risk, particularly as the, I mean well what options do they have open for assessing that risk?” [P31, M, intender, degree+] |
|  | Healthcare professional involvement instils confidence | “No. Again, I’d have no problem with that. If you’ve got a professional medical, and it also ensures the test is done properly as well then. Yeah, it would reassure me I suppose is the word, the best way to- if I was doing the test correctly, yeah.” [P1, M, intender, <degree]  “I have real trust in the health service and my GPs are very good. The nurses are very good. I mean I have bloods taken every now and then for cholesterol and that kind of thing and they’re very good. I have no worries at all about that.” [P33, M, intender, degree+]  “the swallowing is something I do. Whereas, the yanking is something somebody else does.” [P9, M, intender, degree+] |
|  | Confidence that the NHS can cope with capsule sponge screening implementation | “My GP is well up on new things, he’s willing to try new things so yeah, I would be happy enough going to him.” [P27, W, intender, degree+]  “I have friends who bemoan trying to get GP appointments. I don’t have a problem getting a GP appointment… I never have had. My wife goes to a different practice in the same building and she struggles to get appointments but I’m-I seem to have landed on my feet with my GP.” [P33, M, intender, degree+] |
|  | Healthcare professional demeanour is essential for capsule sponge screening | “Not- As long as I felt as though I was confident in the person that was doing it, and the procedure, and the place looked okay, I’d be fine about it. [when asked what she meant] … Well, I suppose their manner.” [P18, W, intender, degree+]  “You know, you’d have nurses who are sensitive and calm, and you’d have nice classical music going… just to make it as relaxed as possible…. I would hope that if a test were offered by a GP there would be some training for the GPs or the nurses or whoever. But no, you'd just want to know that they are proficient at doing it, and I'm sure they would be if they, you know, somebody showed them how.” [P23, W, intender, <degree]  “You go to the nurse at the doctors, so nice. Take their time, talk to you, whatever. That’s the kind of thing you want, not somebody that doesn’t want to be there and lets you know it.” [P27, W, intender, degree+] |
| *Management of Barrett’s* | Barrett’s diagnosis would lead to behaviour change | “Yeah and if there was a way of treating it then you look towards that and it might reduce a lot of stuff. So it would be really helpful in a way to” [P7, W, non-intender, <degree]  “I’d try to [change diet in response to a diagnosis of Barrett’s]. I’ve got a very sweet tooth, so I’d have to try and curb that a bit more. I mean I’m trying to curb it now but it’s hard work. It’s turned me in to diabetic as well. You know, everything that I can do to help myself is going to make a difference.  But I think it would do me good. It might be one thing that would help me to curb some of my sweet tooth.” [P15, W, intender, <degree] |
|  | Enthusiasm for endoscopic monitoring | “ The more monitoring you do, surely it’s a positive thing, yeah? … But to me, it’s very straightforward. If you’re being monitored on a regular basis, surely that has to be to my benefit and maybe the results might not be what I want, yeah? But that’s beside the point. And I just think it’s got to be positive that that’s the case, that actually you are being checked. You are being looked at and that- you know. If things are wrong, then at least you know what condition you’ve got.” [P26, M, intender, degree+]  “if Barrett’s is some condition that they think they should be monitored and I have it unknowingly and I’m not being monitored then to me that's not a good thing. So I would like, to rectify that by finding out if I have got it and therefore being monitored you know, because it sounds like a fairly serious condition that you'd want to know that you have it if you have it, yeah.” [P6, W, intender, degree+]  “ongoing monitoring would be a really nice comfort blanket, but it wouldn’t be anything that I would look back on and think damn, I wish I hadn’t done that.” [P11, M, intender, degree+] |
|  | Oesophagus is an internal organ so monitoring valued | “But it would give you an insight to what’s going on inside your own body, yeah? What’s going on inside your windpipe or your stomach or whatever it may be?” [P1, M, intender, <degree]  “I won’t say there's no cancer cells inside me because I don’t know.” [P5, W, intender, <degree]  “I think any sort of testing is better. It lets you know what’s happening in your body which sometimes you might- well with that poo one, I didn’t feel any different when it was a higher expectation for the doctor. I didn’t feel any different. I wouldn’t have known if it had been a more negative result.” [P29, W, non-intender, <degree] |
|  | Recognising responsibility to attend appointments | “I suppose the only thing is just probably having to travel somewhere to do it. That would be it really. You know. Just having to organise that, to get yourself somewhere to get monitored but otherwise, no not really. I don’t really. I can’t really think of a sort of rational reason why I would even contemplate seeing it as a negative thing.” [P1, M, intender, <degree]  “if you could have it at the GP surgery by the nurse, that would be the best thing for it. That wouldn’t stop me going into the nearest hospital, which would be [CITYB], to have it done, I mean, that would be fine.” [P20, M, intender, <degree]  “I’d have to ask somebody to pick me up or something which is fine. I wouldn’t mind staying in the hospital for a couple of hours if that’s what they meant or anything because for me to the local hospital, well there used to be a bus. It took about an hour to get there but the bus journey now is two buses and it takes a long time. I don’t mind that but I guess if I’m sedated, it will take a long time for me to get over that. I mean I wouldn’t drive, definitely not. I’d have to come back on public transport otherwise. But no, that would be fine.” [P26, M, intender, degree+] |

Theme 2: Coping with uncertainty

| **Concept** | **Support quote [participant characteristics]** |
| --- | --- |
| Wanting to know what is causing symptoms and how to resolve them | “And I think that the crux of it for the vast majority of us is if we could find out what caused it, it would be far nicer to say right, don’t eat, I don’t know, artichokes or whatever but there doesn’t seem to be one thing.” [P11, M, intender, degree+]  “For me personally, if somebody could say to me, right avoid eating, I don’t know, avocado on a Wednesday after eight o’clock, I’d go oh fantastic, I wish I’d known that before. I suspect it won’t be that, I suspect it will be a combination of foods, a combination of how tired I am.” [P11, M, intender, degree+] |
| Drawing comparisons with endoscopy as a barometer for capsule sponge screening | “It looks like it’s less invasive than the camera … So it’s not as bad as it- It’s probably not that bad compared to something, you know, like the camera. The camera seems to be worse to me but that’s probably initial, you know, thoughts… I don’t know. Maybe it’s just the fact of having something that’s mechanical or technical going down, yeah? Rather than something that looks quite innocuous to be honest, in terms of what it looks like. Aesthetically, it looks easier. Maybe sometimes it’s in the mind. You know. What you see in the eye can affect what’s in the mind sometimes.” [P1, M, intender, <degree]  “It’s going to be a little bit uncomfortable but not as uncomfortable as the one I had last year where they put the tube and the camera down your throat. So it’s going to be easier than that. If it’s easier than that then I would have absolutely no problem. It’s literally a few minutes, isn’t it? It’s not going to take very long at all.” [P25, M, intender, <degree] |
| The small possibility of cancer warrants the discomfort of screening | “So even though those figures make it a very low likelihood I would, if it was formally offered, I would go for it. Because it doesn’t, it’s a fairly non-invasive kind of test and I can imagine it being a bit uncomfortable for a short time.” [P40, M, non-intender, degree+]  “It does sound pretty horrible but I think needs must, isn't it? If I thought there was a danger to me, I would have it. Even though I didn’t want it and like it. But I would rather know if there was something wrong with me than be frightened of a test. I would just bear it. Yeah.” [P4, W, non-intender, <degree] |
| Recent attendance at endoscopy diminishes the perceived importance of capsule sponge screening | “I recognise Barrett's, I just don’t feel it is a risk for me, but maybe it is, because obviously I’m- This endoscopy showed I’ve got stomach erosion, which from my understanding is the next stage from gastritis, so, therefore what I’ve had before hasn’t really treated the gastritis. So, am I more at risk, I don’t know. But the reassurance is that they did check the upper oesophagus, mid oesophagus, I’ve got pictures of it and distal oesophagus. So, I know everything’s okay at the moment.” [P13, W, non-intender, degree+] |
| More interested in managing symptoms rather than potential health threats | [when asked how she would feel if he got a negative capsule sponge test result] “Well, what else is it? Is there anything else going wrong down there? And what's causing this pain.” [P5, W, intender, <degree]  “But again, I think I’d still wonder what’s causing the symptoms. I’d probably be thinking; well does that mean I’ve got a life of Omeprazole ahead of me with all the potential-? And it might be okay now but is it going to be the same in five years’ time?” [P2, M, intender, degree+] |
| With increasing age screening may not be a priority anymore | “And I think as you get older- I mean I don’t know what age you are but, as you get older your ability to kind of consider these things as being not really sort of something that you want. You don’t really want the anxiety” [P36, W, non-intender, degree+]  “Maybe as you get older, your attitudes to things change a bit, I guess. I don’t know. I mean, at this moment I would say, yes, I would do it, but maybe if I got a bit more frail or whatever. Because as you age, things happen to you. I don’t know. I mean, when my mother was 70, she was fine, but she got dementia. I can’t remember when it started, but maybe if that happened to me, I may not want to do it anymore. I wouldn’t be able to do it anymore. So unless something like that happened to me, or I physically couldn’t do it for some reason or other, I would carry on, I think.” [P14, W, intender, degree+] |
| More complicated medical histories were more adverse to the potential uncertainty capsule sponge screening creates | “So that is a scary part and because I’m on my own I, I’ve got good friends and everything, wonderful friends but just, it's bad enough sitting here on your own when you haven’t got nothing but when you, you start thinking, oh no what next? You know, and for the last two years have had such a lot of knock-on effects yeah, I don't want no more… I don't know it's, when it's to do with your throat and it could affect your speech, it could you know affect your lifestyle that scares me as much because of not being able to chat.” [P7, W, non-intender, <degree] |
| Wanting Barrett’s to be removed or treated | “So if it’s something like cancer then obviously, you’ll want it dealt with as quickly as possible, yeah? If there’s a foreign body in your body so to speak, yeah? Or a negative thing in your body, again human nature. You would want it removed as quickly as possible, or within as reasonable a time as possible because we all know that often these things do sort of go around time, how long you’ve had something or how long you’ve- if it’s not been detected before, all this kind of stuff.” [P1, M, intender, <degree] |
| Assumption that Barrett’s would be treated surgically or medicinally | “so I imagine to check the state of the gullet in people with long-term reflux to see if there are any indicators of potential problems and get it early enough if there are. Presumably the test, when the sponge goes off to the laboratory, the test that they do on it will have a means of identifying indicators of cancers, cancer, and then something swings into action.” [P23, W, intender, <degree] |
| Motivation to extend life | “I think as you get older mortality- I’m early 60s and not early 80s, if I was early 80s I’d probably think different about this, if I get that far. But, at my age now I have had colleagues, counterparts that have passed away. So, it’s not something I’m dwelling on every day, but it is an awareness that mortality is something that will catch us all eventually.” [P11, M, intender, degree+]  “if you can do something that will prolong your life, I can’t see why you wouldn’t do it.” [P2, M, intender, degree+] |
| Concern about sponge detachment | “it looks like a tea bag almost and they break quite often, so, yeah, that’s a concern. It depends what the rate is I suppose, you need to be able to give you anything from one in so many thousands you perhaps wouldn’t worry about it so much. But if one in every 50 is breaking, then I’d be a bit concerned, yeah.” [P20, M, intender, <degree]  “that’s when I kind of thought oh God that comes up quick what if the string broke.” [P35, W, intender, <degree] |
| Inability to undergo emergency endoscopy making capsule sponge screening futile | “And we take it for granted that we can move our head to the left and the right easily, I can’t do that, I’m fixed… So, an endoscopy you have to have your head to the left and on your side and I can’t get in to that position or do that. If they did it under an anaesthetic then I would need support after that to put me back together again, because they would have to force my head over to the left. Because I’ve got those three- I’ve got those discs there that are stopping it because I’ve got no sponge between the discs, so, they’re held fast… [ when asked what she would do if offered the test] … Well I’d decline it because I can’t even turn my head to the left. And I’d wonder if it did go wrong how the heck they would then do an emergency endoscopy on me.” [P10, W, non-intender, <degree] |
| The importance of test accuracy to make the discomfort it causes worthwhile | “It’s not natural having something shoved backwards down your throat, or into your stomach maybe. Then you’d want to have the most accurate results coming out of that if you go through that process…**.**I think if that was the case, then I think I’d rather go through the process with the camera.” [P1, M, intender, <degree]  “And I suppose you would want to know how efficient it was, you know, how good it is. If I’m going to go through hell and high water is this worth going through. How reliable it is.” [P12, W, intender, degree+]  “At the moment, because it’s only a testing stage, I might think well why put myself through the trauma of doing it when actually it’s not really going to make a lot of difference to picking up these issues.” [P24, W, non-intender, <degree] |
| Preference for endoscopy due to perceived accuracy | “I think I’d rather go through the process with the camera. I think it’s about- What you want is, you want- Nothing’s certain, I understand that but you want a degree of certainty around- If you’re going through a procedure, you want there to be a certainty around that. That sort of matches the level of discomfort you may or may not experience in this case.” [P1, M, intender, <degree]  “if you’ve got a camera down there, they can actually see what’s going on. If you take the tablet that you are suggesting, maybe something could be missed… They can actually look, can't they? They look and see what's going on, and they can see if they need to take biopsies. I mean, yeah, you'd obviously be concerned about your health, and if you were that concerned, you'd want to be reassured that there's nothing going on.” [P4, W, non-intender, <degree] |
| Aversion to something being missed in the instance of false negatives | “Because it’s new I think there would be a lot of questions in terms of like the reliability of it. For example if the sponge is pulled up- Okay, so you go for a smear test, if you have a smear test sometimes they don’t get enough cells, is this likely to happen with this, that would be my first question.” [P36, W, non-intender, degree+]  “It’s kind of, there's just no point having it if it’s not accurate, is it? Is it accurate enough? Could something be missed?” [P4, W, non-intender, <degree]  “I don’t know. I mean obviously it’s only at the testing stage at the moment so I think maybe once it had been proven that it was really beneficial, that may make me feel different. At the moment, because it’s only a testing stage, I might think well why put myself through the trauma of doing it when actually it’s not really going to make a lot of difference to picking up these issues.” [P24, W, non-intender, <degree]  “So if they’re putting a sponge down, is it collecting samples all the way up or just in one area? I mean if you’ve got cancer in a different part to what it’s collecting, is it going to collect the whole way? Yes, or is it going to just take from one bit and it could be in a different part that you’ve got this Barrett’s.” [P27, W, intender, degree+] |

Theme 3: Proving yourself as a worthy candidate for capsule sponge screening

| **Concept** | **Support quote [participant characteristics]** |
| --- | --- |
| Severe or long-term symptoms that are difficult to control make participants feel the test is relevant to them | “For me, I think because I’ve had acid indigestion for a long time and I suspect my throat is damaged, I suspect my larynx is damaged, I would see it as a bonus I think to have a test like this and have some confidence that any issues I might have might be detected earlier.” [P2, M, intender, degree+]  “Oh I’d like to have it, that's. that you know, seems to me would be a good thing to have given that I’ve suffered so badly with reflux in the past yeah, yeah I’d like to try and have it actually yeah.” [P6, W, intender, degree+]  “I would be willing to accept that maybe for the period of time that I wasn’t, that I didn’t, that I wasn’t controlling my reflux, that, you know, it’s possible that some kind of damage could have been done during that time,… So, you know, I'd be more than happy to do a test just to check, yeah.” [P22, M, intender, <degree] |
| Capsule sponge screening would be good for others | “Aye, definitely. But I would advise anybody else, younger people to go… Because they’ve got a longer life and I feel it’s better for them. Like my grandkids and things like that, I would say to them.” [P30, W, non-intender, <degree] |
| Feelings of risk due to age and family history being used to justify candidacy | “I can give you an example, I’ve just gone back to Scotland last week my wife and I to see my sister who has got cancer. And she only is eleven months older than me my sister, so they are eleven months older than me, my dad died of cancer. Now my sister has got cancer. It’s always on my mind, you know what I mean. It’s always on my mind.” [P42, M, intender, <degree]  “Yeah. One of my uncles had throat cancer a long time ago. Yeah, so- Yeah, it’s quite a worry, yes.” [P19, W, intender, <degree]  “But at the same time though, you know, as you get older, you get more chances of things like cancers. You get more chances of terminal diseases, yeah, and it’s just something that I’m aware of.” [P1, M, intender, <degree]  “I think it’s important as well because if you want to live a long life, you’ve got to make sure that, at my age anyway as you get older, you have these tests and make sure things are diagnosed early and treated early so that it doesn’t become more serious later on.” [P26, M, intender, degree+] |
| Perceptions that the NHS is under a lot of strain | “I’m very aware of NHS waiting lists. I’m very aware of stuff like that. I’m very aware of priorities and things like that. So I know sometimes it’s- It’s just- the processes don’t always happen in the timescales they should do.” [P1, M, intender, <degree]  “Well, as the waiting list gets longer, the problem gets worse, doesn't it? That’s the hue and cry about any cancer thing on the NHS, isn't it? You start with a headache and end up with migraine [laughs], but if you could see the hospital quicker, then again, something can be done about it.” [P5, W, intender, <degree] |
| Having to fight for the care they feel they deserve or outsourcing to private services | “And I was triaged as urgent on the referral system, but the waiting list is 29 weeks plus. So, I just did what I do I made some phone calls and found out that [HOSPITAL NAME] they do- NHS has gone private. So, I then, armed with that, I phoned my doctor and said right can you do me a referral to [HOSPITAL] and that’s what they did and that’s how I got to see them within a month, and they were lovely.” [P10, W, non-intender, <degree]  “So, I then lived on Gaviscon and I’d taken out Bupa so I had private. So, I went and had a gastroscopy a first one and he said it’s appallingly inflamed and I’ll pass you on to a surgeon, and I had the first fundoplication and that was fine, that was fine, that was in 2001… Then I had another gastroscopy and I went to see a physician who said I should be having the gastroscopies every three years because of the inflammation… The physician who was doing this three yearly said look this is ridiculous you’re paying a load of money and Bupa wouldn’t pay for them. So, go to [HOSPITAL]. So, I went to [HOSPTIAL] and I had a couple there and then the last one, he went oh it’s all right and you’re discharged. So, that’s how it’s left.” [P12, W, intender, degree+] |
| Concern about not being able to complete the procedure | “I just hope I can swallow it, and I don’t gag. For your sake, rather than mine” [P5, W, intender, <degree]  “as far as I can see, the issue here would be how easy would it be for me- and that’s me personally, not anyone else, to swallow that new thing. Now, it could be that- obviously, I can swallow bits of food that are bigger than that. Obviously, and do. That and the string. Without trying, I don’t know.” [P9, M, intender, degree+]  “I have a very bad gag reflex, so when I go for gastroscopies they go do you want sedation and I go- I proffer my vein and say there is nothing about this I wish to remember. So- I have trouble cleaning my teeth at night sometimes, putting the toothbrush in my mouth I start gagging so the dentist they tried doing it on a dry brush. So the thought of trying to swallow that just turns me over to be perfectly honest.” [P12, W, intender, degree+] |
| Aversion and anticipated embarrassment due to gagging or vomiting | “Because I was terrified at the feeling of it going down that I would gag. Well actually do you fast before the test, I can’t remember that bit? That’s grand so there’s very little chance of me actually producing vomit all over the place? Because I would find that terribly- Okay I would deal with it but I would find that very embarrassing.” [P35, W, intender, <degree]  “I know because there’s something that I’m thinking- Just the thought of it. Because I hate being sick. I really, really hate being sick.” [P17, W, non-intender, degree+]  “I suppose, yes. Obviously because the- Obviously, when you’re retching anyway, if you’re being sick it’s horrible.” [P19, W, intender, <degree] |
| Feeling that they would be depriving someone else of a test if they were to accept the offer but then unable to complete the procedure | “Because again I don’t want to waste anybody’s space or time in a queue fora test.” [P38, M, non-intender, <degree]  “Yeah, I guess they’re not cheap and I wouldn’t want to waste it as well. You know, getting it in my mouth and then not being able to swallow it or immediately puking it back out would be such a waste of money.” [P12, W, intender, degree+] |
| Previous mention of cancer during consultations reinforces candidacy | “Well I’m just going back in to the room where I was with the consultant and she said about Barrett's and she said about oesophageal cancer. So, that was planted in my head, you know, so I’d got that already in my head because she’s mentioned it” [P10, W, non-intender, <degree]  “This whole reflux thing does make me slightly uneasy because it’s something that I can’t control. .. I think it’s quite strong, yeah. I think I would- Well, when I was telling you earlier about the time I went about four years ago, yeah. Obviously, that was quite strong in my mind, yeah? The doctor didn’t reassure me with her initial phone call to me, I have to say. Yeah, but- you know. Basically, I am quite aware that it’s something that can happen, yeah with reflux.” [P1, M, intender, <degree] |
| Wanting to make lifestyle changes following a Barrett’s diagnosis to make it worthwhile | “So I suppose I’ve got a similar sort of gung-ho attitude to this. If you knew that your acid reflux was doing you some actual harm, then it might make you alter the way you behave in terms of what you eat. Even things like- I mean even things like putting a couple of books under the legs of your bed to put your bed at an angle can make a difference to your acid reflux. I’ve not done that because I share a bed with my wife and she’d probably be grumpy that she’s sliding to the bottom of the bed. But there are certainly things I know I could do now that would reduce my acid reflux, but it’s much easier just to take a Rennie or a Gaviscon in the evening and suppress it with that, or go onto Omeprazole when actually there’s probably still- I could probably cut out bread entirely because people do and they say that helps but I still have a sandwich every day at work.” [P2, M, intender, degree+] |
| Wanting to be proactive if diagnosed with Barrett’s | “Well, prevention rather than cure. Hopefully they will say that there's nothing wrong with you, therefore that’s one thing off the list, you know? You're okay, carry on. And if you’re not okay then hopefully something positive can happen.” [P5, W, intender, <degree]  “If there’s something wrong with me, I’d want it fixed as quick as possible.” [P2, M, intender, degree+] |

**Supplementary material 8: Patient and Public Involvement:**

One PPI collaborator was involved in the grant application and contributed to the original conceptualisation of the wider project. Three PPI contributors were consulted during the study set up to ensure that the materials were appropriate, sensitive and understandable, and provided feedback on the interview process. The researchers met with PPI representatives via Microsoft Teams to discuss study documents and procedures. Prior to these meetings, the representatives were sent documents and questions to review.

PPI representatives provided feedback on the study procedures, topic guide and documents (consent form, information sheet, and screening questionnaire). One contributor participated in a pilot interview. PPI feedback led to changes in the wording of materials, the information provided in the interviews, and the way that questions are worded within interviews. PPI feedback was essential in deciding the design of the study (interviews vs focus groups) and to highlight areas to focus on in the topic guide and sensitive topics that should be approached carefully.

Patient and public involvement strengthened this study, but on occasions, it was difficult to reconcile opposing opinions between representatives, and instances where best practice and research evidence opposed the opinions of PPI representatives. In these instances, the wider research team met to discuss the best course of action, and additional PPI representatives were consulted to further our understanding of key issues, such as reflux stigma and risk awareness.”

**GRIPP2 Short Form checklist:**

| Section and topic | Item | Section in which reported |
| --- | --- | --- |
| 1: Aim | Report the aim of PPI in the study | Appendix |
| 2: Methods | Provide a clear description of the methods used for PPI in the study | Appendix |
| 3: Study results | Outcomes – report the results of PPI in the study, including both positive and negative outcomes | Appendix |
| 4: Discussions and conclusions | Outcomes: Comment on the extent to which PPI influenced the study overall. Describe positive and negative effects | Appendix |
| 5: Reflections / critical perspective | Comment critically on the study, reflecting on the things that went well and those that did not, so others can learn from this experience | Appendix |

**Supplementary material 9: Suspected fraudulent participation:**

*“*W*e* encountered a large issue surrounding fraudulent participation. In this study, we define fraudulent participation as: “individuals that contact a research team to take part in a study and give false responses” (1). Often, this seems to be driven by financial incentive (1), but some believe it can also be a malicious attempt to threaten the integrity of research data (2).

In this study, we believe participants were reporting false information in the pre-screener questionnaire in order to meet the study eligibility criteria. This included: UK residency age, and reflux symptom occurrence.

I interviewed one participant that I believed was ‘fraudulent’ so removed this participant’s data from analysis. I also later identified several other suspected fraudulent participants that had completed the pre-screener questionnaire, who I decided not to invite for an interview.

My first suspicion came after conducting an interview. The interview was conducted on Microsoft Teams and the participant refused to turn their camera on. Throughout the interview, the participant spoke about their reflux in a strange way that seem different to the other 15 interviews I had conducted up to this point. They often spoke about reflux in a hypothetical way such as ‘people that have reflux have symptoms including…’ and I got the impression that they were not speaking from genuine experience. For example, in other interviews, participants described their medication by drug or brand names e.g. Omeprazole and Gaviscon, whilst this individual described their medication only in generic terms like ‘proton pump inhibitors and ‘histamine’ medications. Furthermore, the participant often delayed answering questions and would ask for them to be repeated. Whilst I cannot be certain, I believed I heard some typing and talking in the background during the call and suspect that the participant was asking for the question to be repeated as a stalling technique to allow them time to look up the answer. When I later asked ChatGPT to answer some of my questions, very similar responses to those given by this participant were generated, including the use of generic medication terms.

Following the interview, I reviewed this participant’s pre-screener questionnaire responses and noticed that the participant had provided a telephone number using an area code that does not exist in the UK, and that they stated they were living in Glasgow, where I had not yet started recruitment. The address that they listed was in the centre of Glasgow where there are mostly retail units, meaning it is unlikely (although not impossible) that he was actually living there. Furthermore, the email address that he used was in the format of ‘FIRSTNAME_LASTNAME_123@gmail.com where the numbers at the end were a random sequence of 3 numbers. The use of free email accounts and addresses in this format has been identified as a red flag for fraudulent participation in other studies (ref?). The final ‘red flag’ identified was that the participant stated that they were recruited through Twitter, which at the time was not a way that I had been approaching participants. When I asked this participant for an address to post their voucher to, they did not provide an address and instead asked for the voucher to be sent via email. They requested an Amazon or ‘love2shop’ voucher, which has again been reported as a red-flag in other studies, since these vouchers can be redeemed anywhere in the world (1).

Following this experience, the project team decided it was best to exclude this participant to maintain data integrity. I conducted some research and identified guidance which helped me to identify further red flags. At this point I had already recruited several other participants that I subsequently realised were most likely fraudulent following this interaction. This included: spelling errors in names/email addresses, the consistent ‘FIRSTNAME_LASTNAME_123’ email format, unlikely addresses (including 221B Baker St, 10 Downing Street and Buckingham Palace), inaccurate telephone numbers, including landline numbers for areas different to those reported in the address, and non-existent telephone area codes. Finally, there were several instances where suspected fraudulent participants reported they had heard about the study through methods I had not used including ‘through a physician’ and various social media channels.

For the interviews already scheduled that I suspected to be fraudulent, I went through a process to verify authenticity. This included asking for a mobile number and scheduling a ‘pre-interview’ call. Often suspected fraudulent participants would ignore these requests and would sometimes still join the scheduled call for the interview. In other instances, they would reply to request that the call was conducted on Microsoft Teams. In this instance, I obliged and when on the call requested that they turned their camera on. Every time I made this request, suspected fraudulent participants told me that their camera was not working, or that they had turned it on (but it remained black on my screen). I suspect participants did not want to turn their camera on since it might reveal that they were not the age they had reported to be in the pre-screener questionnaire, or because they were hoping to have someone else present to help them think up answers to the interview questions. Where individuals did agree to a pre-interview call, I asked them to verify some information from the pre-screener questionnaire including their home address and telephone number. Participants later classified as suspected fraudulent often gave me inconsistent information that did not match that given on the pre-screener questionnaire or were unable to provide a telephone number or address at all. In these scenarios, I informed participants that we were oversubscribed for people in their demographic groups (e.g. from London, or men aged 65+) as a way to legitimately end the call and their involvement in the interview study.

Going forward, I excluded participants that completed the pre-screener survey and had ‘red flags’ for suspected fraudulent participation. Where I was in doubt, I followed the above procedure of arranging a telephone call to confirm identity. In many instances individuals were unable to confirm their addresses, phone numbers or gave dubious explanations about how they heard about the study. There were some instances where I had suspected a participant was fraudulent, but they were able to answer my questions, and in these instances they were included in the study since I was confident they were a genuine participant.*”*

1.	Mistry K, Merrick S, Cabecinha M, Daniels S, Ragan J, Epstein M, et al. Fraudulent Participation in Online Qualitative Studies: Practical Recommendations on an Emerging Phenomenon. Qualitative Health Research. 2024:10497323241288181.

2.	Teitcher JE, Bockting WO, Bauermeister JA, Hoefer CJ, Miner MH, Klitzman RL. Detecting, preventing, and responding to "fraudsters" in internet research: ethics and tradeoffs. J Law Med Ethics. 2015;43(1):116-33.
